# Quantitative Microdialysis: Experimental Protocol and Software for Small Molecule Protein Affinity Determination and for Exclusion of Compounds with Poor Physicochemical Properties

**DOI:** 10.3390/mps3030055

**Published:** 2020-07-30

**Authors:** Steven Shave, Nhan T. Pham, Connor B. Śmieja, Manfred Auer

**Affiliations:** 1 School of Biological Sciences, IQB3, University of Edinburgh, The King’s Buildings, Max Born Crescent, CH Waddington Building, Edinburgh, Scotland EH9 3BF, UK; s.shave@ed.ac.uk (S.S.); Nhan.Pham@ed.ac.uk (N.T.P.); C.B.Smieja@sms.ed.ac.uk (C.B.Ś.); 2 School of Biological Sciences and Edinburgh Medical School: Biomedical Sciences, University of Edinburgh, The King’s Buildings, Edinburgh EH9 3BF, UK

**Keywords:** label free screening, affinity determination, dialysis, K_D_ determination, promiscuity, aggregation, nonspecific binding, protein binding analysis, chromatography, drug discovery

## Abstract

Quantitative microdialysis is a traditional biophysical affinity determination technique. In the development of the detailed experimental protocol presented, we used commercially available equipment, rapid equilibrium dialysis (RED) devices (ThermoFisher Scientific), which means that it is open to most laboratories. The target protein and test compound are incubated in a chamber partitioned to allow only small molecules to transition to a larger reservoir chamber, then reversed-phase high performance liquid chromatography (RP-HPLC) or liquid chromatography–mass spectrometry (LC–MS) is used to determine the abundance of compound in each chamber. A higher compound concentration measured in the chamber that contains the target protein indicates binding. As a novel, and differentiating contribution, we present a protocol for mathematical analysis of experimental data. We provide the equations and the software to yield dissociation constants for the test compound-target protein complex up to 0.5 mM K_D_, and we quantitatively discuss the limitations of affinities in relation to measured compound concentrations.

## 1. Introduction

**Scheme 1:**
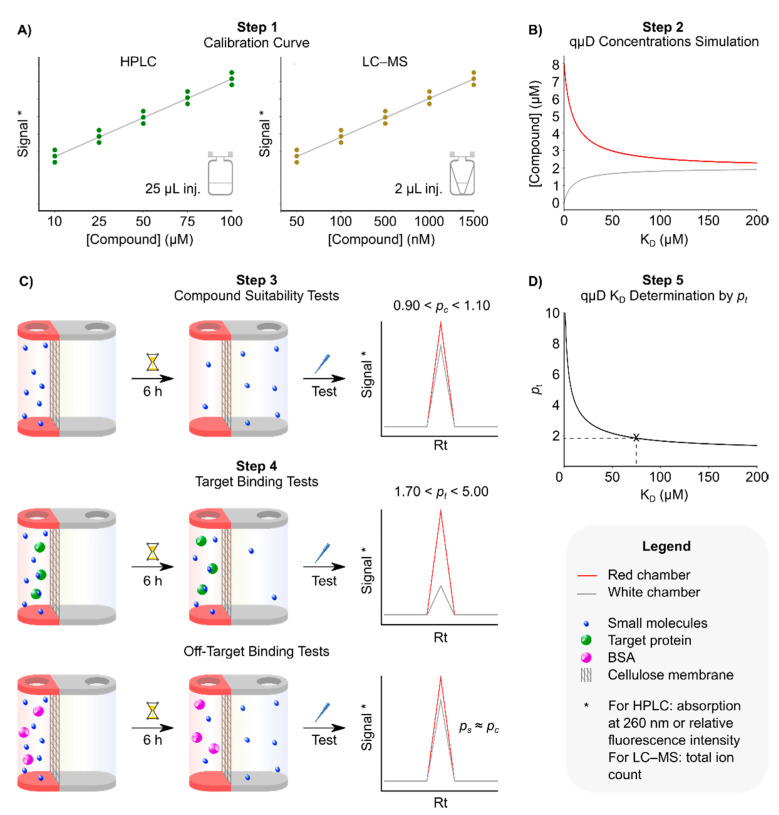
Schematic representation of the quantitative microdialysis (qµD) process. Step 1—Determination of the detection sensitivity of test compounds by reversed-phase high performance liquid chromatography (RP-HPLC). Small molecules are monitored either by absorption at 260 nm, or by relative fluorescence intensity at the appropriate, predetermined excitation or emission wavelengths. If LC–MS is used for quantification then the ionisation mode and sensitivity is monitored, and the small molecule peak is resolved by the total ion count. Step 2—Identification of the appropriate target protein concentration. Knowing detection limits and compound solubility, response (compound concentration) can be simulated over a range of K_D_s to ensure detection is possible at the desired K_D_ range. Step 3—A compound-only experiment is performed to test whether the small molecule is suitable for a dialysis experiment at all. The partition coefficient of the small molecule in the absence of protein (*p_c_*, where the ‘c’ denotes ‘control’) needs to be between 0.9 and 1.1. If the partition coefficient is higher, the compound is suggested to be excluded. Step 4: The identical experiment as in Step 3 is repeated, including the target and control proteins in concentrations simulated and predetermined in Step 2. Bovine serum albumin (BSA) is given as an example. RP-HPLC or LC–MS analyses now lead to the partition coefficients both in the presence of the target protein and off-target proteins (*p_t_*, where the ‘t’ denotes the ‘target’, and *p_s_*, where the ‘s’ denotes ‘selectivity’). Step 5: determining the K_D_ of the target–ligand complex using concentrations or *p_t_* values.

Quantitative microdialysis (qµD) is a traditional, yet powerful technique for the analysis of two important properties of small molecules of interest [1,2,3,4]: (a) the diffusability of small molecules, which is affected by their intramolecular aggregation and their surface binding properties, and (b) the determination of their affinities to target proteins, using equipment found in many laboratories. Conceptually, dissolved test compounds may freely move between two chambers partitioned by a semipermeable membrane that allows only small molecules to pass through. Along with these freely moving compounds, one chamber also contains either the target protein (to which it is assumed or known to bind to) or one or more control proteins (which serve to test the selectivity of the target protein binding reaction). A thermodynamic equilibrium reached in the absence of a protein after a certain incubation time should result in equal concentrations of free compound in the two chambers. If the compound engages in nonspecific binding towards the material of the chambers or the separating membrane, different compound concentrations will be detected between the two chambers, using any of the suitable analysis techniques. In the presence of a target protein binding to the test compound, the two chambers are also expected to contain different compound concentrations (see Scheme 1).

Analysis of the compound concentrations in each chamber by RP-HPLC or LC–MS will reveal a higher abundance of the compound present in the protein-containing chamber if the compound binds favourably to that protein. The signal detected represents the sum of the protein-bound compound, and free compound. From these concentrations, the amount of compound bound to the protein can be deduced. Then, since the total amount of protein is known, the binding affinity of the complex can be calculated. Many highly sophisticated techniques have been developed during the last decades to analyse and quantify the interactions of small molecules with proteins, including multiple modes of fluorescence spectroscopy [5], nuclear magnetic resonance (NMR) [6], mass spectrometry [7], affinity selection [8,9] and size exclusion chromatography [10], just to name a few. We apply all of the above in our hit and lead identification projects. The power of the qµD technique lies in its applicability to unlabelled and labelled compounds and proteins, the ease with which it can be set up, and its cost effectiveness. Very importantly, a substantial amount more about the properties of the small molecules can be revealed compared to other techniques, and very low affinity interactions up to K_D_s of 500 µM can be determined reliably. These advantages make qµD an integral component of our label-free affinity screening platform (LFAP) [11], which is also compatible with fragment-based screening and quantification. Here, we present the experimental and computational design of the qµD process and provide a detailed protocol and standard operating procedure (SOP) for using our software for K_D_ calculation.

## 2. Experimental and Computational Design

### 2.1. Experimental Design

Step 1 of our qµD process is the determination of the appropriate concentration of the small molecules needed for detection. Non-fluorescent or fluorescently labelled compounds can be analysed by RP-HPLC. For non-labelled compounds, a concentration series of 10, 25, 50, 75 and 100 μM is recommended, with 25–50 μM usually falling in the middle of the linear range. Our lab used this method to confirm hit compounds arising from on-bead screening [12]. Standard library compounds or fragments that do not contain aromatic components cannot be detected by RP-HPLC in sufficiently low concentrations for the qµD process. Instead, these compounds were analysed by LC–MS. To find the appropriate concentration in this case, it must first be established whether the compounds ionise at all, and in which detection mode. The LC–MS method for concentration determination was detailed in Section 3. This protocol was established for analysis by a Thermo Fisher^TM^ LTQ Orbitrap XL^TM^. Adaptation of the protocol to other mass spectrometry instruments should be straightforward for any lab proficient in mass spectrometry. In Scheme 1 (Step 1), the concentration range comprising of 50, 100, 500, 1000 and 1500 nM, with 250–500 nM being in the middle of the linear range for most compounds, was derived from an Orbitrap instrument. Step 2 of the qµD analysis process involves simulating the dialysis process. This will inform the experimentalist as to the most suitable concentration of the target protein, to maximise the disparity of compound concentrations between the two chambers, within the detection limits. Therefore, this step is critical to maximise the accuracy of the method. Step 2 was described in more detail in Section 2.2. Step 3 involves testing whether the compound equilibrates uniformly enough between the two dialysis chambers in the absence of the protein, to make it suitable for incubation with the target protein and one or more negative control proteins in Step 4, to test the compounds potential for unselective or nonspecific binding. It is well known in biochemistry that proteins need to be tested for their folding state and aggregation potential. On the other hand, compounds contained in screening libraries, and—more importantly—primary screening hits, are not as routinely investigated for inherent problematic behaviours. These behaviours often lead to false positive and false negative results. Dialysis offers a versatile way to address this; it can be used to test surface binding as a function of buffer composition, pH, additives and other assay variables, as well as to identify dynamic aggregation and promiscuous binding as a function of concentration. Using the predefined compound concentration, *p_c_* is determined. This should ideally be below 1.10 after an appropriate time period in the target incubation step. Now, having simulated the concentration of the target protein, and proven the purity and suitability of the test compound, Step 4 of the qµD process may be performed. In Scheme 1 (Step 4), a single incubation experiment with the target protein and one with BSA as a control is shown. Additional selectivity and specificity controls can be integrated as needed. The time frame for equilibration can vary as a function of the concentration of the compounds, affinity to the target or temperature, however a study by Walters [13] demonstrates equilibrium of 18 diverse drugs covering a wide range of physicochemical properties is achieved within 6 h. Similar studies by van Liempd et al. [14] and Ye et al. [15] further demonstrate 6 h to be an adequate equilibration time. We started with 6 h of incubation, defined by the compound-only experiment. To determine the partition coefficients, the compound concentration needs to be analysed in both compartments. A high concentration of protein can interfere with the chromatographic separation, making it desirable to remove the protein prior to analysis. To this end, methods for protein denaturation and precipitation using organic solvents, acid or heat have been developed [13]. However, we have found that coprecipitation of the compound with the target protein may occur to a certain extent, in line with observations by Vanholder et al. [16], reducing the reliability of the subsequent measurement. Furthermore, adding the denaturation agent to the sample results in a large volume increase, which need to be offset and the original volume established to perform a quantitative analysis. Hence, we present here a method where no protein precipitation or chromatography of the protein containing samples is needed to analyse samples by RP-HPLC. In addition, for the MS analysis, only a simple dilution is required (due to the sensitivity of the mass spectrometer used), which does not compromise the sample quality. At this dilution it was found that the amount of protein present was compatible with our LC–MS setup and therefore analyses could occur without interfering with the compound detection.

### 2.2. Materials

- Self-sticking plate seal (Sigma-Aldrich, Cat no.: Z369667-100EA);
- Rapid equilibrium dialysis (RED) device reusable base plate (Thermo Fisher Scientific, Cat. no.: 89811);
- RED device inserts, 8.0 kDa (Thermo Fisher Scientific, Cat. no.: 89809);
- RED device insert removal tool (Thermo Fisher Scientific, Cat. no.: 89812), or a pair of non-pointy tweezers;
- Assay buffer. A buffer that is compatible with the target protein;
- 20% ethanol. Made from ethanol (Fisher Scientific, product code 10437341, purity ≥ 99.8%) and Milli Q water;
- Pluronic F127, low UV absorbance, (Thermo Fisher Scientific, Cat. no.: P6867);
- Dimethyl sulfoxide (DMSO) anhydrous, (Sigma Aldrich, Cat. no: 276855, purity ≥ 99.9%;
- Formic acid (FA) for LCMS LiChropur, (Sigma Aldrich, Cat no.: 5.33002, purity 98–100%);
- HPLC grade H_2_O and Acetonitrile. H_2_O: Milli Q water with a resistivity ≥ 18 MΩ cm. Acetonitrile HPLC Plus (Sigma Aldrich, Cat. no.: 34998, purity ≥ 99.9%);
- Trifluoroacetic acid (TFA) for HPLC, (Sigma Aldrich, Cat. no.: 302031, purity ≥ 99.0%);
- 1.5 mL Eppendorf safe-lock tubes (Eppendorf, Cat. no.: 0030120086) or 1 mL glass vials (VWR, Cat no.: 548-0385, and lids, Cat. no.: 548-0389);
- Chromacol glass insert vials, (VWR, Cat. no.:548-0260);
- Insert vial snap caps (VWR, Cat. no.:548-0265);
- Standard laboratory orbital shaker, such as Ika Schüttler MTS 4 S 2 Microtiter Shaker.

All reagents were used as received.

### 2.3. Equipment

• Agilent 1100 series HPLC system

RP-HPLC method: RP-HPLC analysis were performed on an Agilent 1100 series HPLC system, consisting of a quaternary pump (G1311A), a degasser (G1322A), a well plate autosampler (G1367A), a fluorescence (FLD) detector (G1321A) and a diode array (DAD) detector (G1315B). The column used was an Agilent ZORBAX SB-C18, 4.6 mm × 150 mm, 3.5 μm particle diameter and 80 Å pore size. Analyses were performed using a gradient of solvent A, H_2_O containing 0.1% trifluoroacetic acid (TFA) and B, Acetonitrile (MeCN; HPLC grade) containing 0.1% TFA with a flow rate of 0.8 mL/min. The gradient used: 0–5 min, 5% B; 5–24 min, 5–100% B, 24–26 min, 100% B; 26–27 min, 100–5% B and 27–30 min, 5% B. HPLC traces were always measured at absorptions of 210 nm, 220 nm, 260 nm and 280 nm. All data analyses were performed using the Agilent Chemstation software v.03.01.

- Thermo Fisher^TM^ LTQ Orbitrap XL^TM^.
- Waters Acquity UPLC^TM^ system.

LC–MS method: All LC–MS analyses were performed on a Waters Acquity UPLC system consisting of a cooled sample manager and a binary solvent manager coupled to the electrospray ionisation (ESI) source of the LTQ Orbitrap via an in-house filled column, 0.2 mm × 120 mm, 5 μm particle diameter and 120 Å pore size (ReproSil-Pur 120 C18-AQ, 5 µm). The solvent system for elution was: A—H_2_O containing 0.1% formic acid and B—MeCN containing 0.1% formic acid. A standard gradient method was used for analysis, which was: 0–3 min, 0.5% B; 3–18 min, 70 % B; 18–22 min, 95% B and 22–25 min 0.5% B; with a flow rate of 8 μL/min. The mass analyser was an LTQ Orbitrap (Thermo Finnigan) with an ESI injection and it was set to record mass data at the start of the gradient. Thermo Xcalibur software was used to analyse the mass spectra and calculate intensities for generation of calibration curves and K_D_ determination from equilibrium microdialysis.

### 2.4. Computational Design-Simulating System Behaviour

The previous microdialysis analysis method presented by Weidemann et al. [12] was based on solving the necessary differential equations followed by simplifications based on the assumption that the protein is in large excess over the small molecule tested. Here we present the derivation of a complete system definition achievable using the symbolic manipulation abilities of Wolfram Mathematica (version 12.1) [17]. Additionally, we included a term to correct for the possible non-complete equilibrium of test compounds. Whilst a strongly and indefinitely non-equilibrating compound (see for reasons above) should be excluded from the assay, the inclusion of this term performs a correction for compounds, which would achieve equilibrium if given enough time. To arrive at the underlying equations governing a microdialysis experiment, we defined three locations in which compound can exist. These are: free in the white chamber (since there is no protein), free in the red chamber (unbound but in the presence of protein) and finally a third virtual compartment–bound to protein in the red chamber. The system can thus be defined by the following equations.

(1) $$\begin{array}{c} l0*\left(whitevol+\;redvol\right)==((lwhite*whitevol)+((lfred+tl)*redvol)),\\ lfred==p_{c}*lwhite,\\ tl*K_{D}==tf*\left(lfred\right),\\ lred==lfred+tl,\\ p_{t}==lred/lwhite \end{array}$$

where *l*0 is the ligand concentration representing an equal distribution of ligand across both sides of the partition between red and white volumes at equilibrium, following an amount of ligand being added to the red volume in the absence of the protein. *whitevol* and *redvol* are the volumes of the red and white chambers, respectively. *lfred* and *lwhite* are the concentrations of free ligand in the red and white chambers, respectively. *tl* is the concentration of target–ligand complex in the red chamber. *tf* is the concentration of free (unbound to ligand) target protein in the red chamber. K_D_ is the dissociation constant of the protein–ligand (target–ligand) complex. *lred* is the overall concentration of the compound in the red chamber, including the bound and unbound compound. *p_t_* is the ratio between *lred* and *lwhite*. *p_c_* is the ratio of the compound concentrations in the red and white chambers in the control experiment in the absence of the protein; ideally this should be equal to 1 as both of these concentrations would be *l*0.

This system of equations for *lred*, *lwhite*, *p_t_* and K_D_ can be solved using Wolfram Mathematica (version 12.1), allowing it to be fully simulated for experimental planning. Three equations for K_D_ determination can be produced using each of the three experimentally determined variables: *lred*, *lwhite* and *p_t_*. This enables the K_D_ to be determined using a single concentration sample from either of the chambers, or by sampling both and determining a *p_t_*. In addition, the Python [18] code can be used to perform the simulation of custom microdialysis experiments, reproduce the simulation plots within this protocol and explore numerous examples; this is freely available under the open source MIT license at https://github.com/stevenshave/microdialysis.

The above equations defining the system may be manipulated by Mathematica to remove unknowns and solve for specific member variables. The following code recreates the system:

(2) $$\begin{array}{ll} model=t0== & tf+tl,l0*\left(whitevol+redvol\right)=\\ & =lwhite*w\mathrm{hitevol}+lred*r\mathrm{edvol},lfred==pc*l\mathrm{white},tl{*K}_{D}\\ & ==tf*\left(lfred\right),lred==lfred+tl,pt==lred/lwhite; \end{array}$$

Which may then be solved for different member variables with intermediate steps of elimination for unknown variables such as *lfred*, *tl*, *tf* and more, depending on the subject of the formula.

(3) $$\begin{array}{c} Solve\left[Simplify\left[Eliminate\left[model,tf,tl,lfred,lwhite,pt\right]\right],lred\right]\left[\left[1\right]\right]\\ Solve\left[Simplify\left[Eliminate\left[model,tf,tl,lfred,lred,pt\right]\right],lwhite\right]\left[\left[2\right]\right]\\ Solve[Simplify[Eliminate\left[model,\left\{tf,tl,lfred,lwhite,moleslred,moleslwhite,lred\right\}\right]],pt]\left[\left[2\right]\right] \end{array}$$

Notice that in the above calls to Mathematica’s Solve function, we took solutions 1, 2 and finally 2, as signified by the square brackets at the end of each line, choosing the correct polynomial root produced in an intermediate step determining equations for 1:1 binding. The direct analytical solutions for *lred*, *lwhite* and *p_t_* are as follows:

(4) $$\begin{array}{l} lred=\frac{1}{2\left(p_{c}{}^{2}redvol^{2}+pcvalue*redvol*whitevol\right)}*(2*l0*p_{c}{}^{2}*redvol^{2}\\ \;+K_{D}*p_{c}*redvol*whitevol+l0*p_{c}*redvol*whitevol+2\\ \;*l0*p_{c}{}^{2}*redvol*whitevol+p_{c}*redvol*t0*whitevol\\ \;+K_{D}*whitevol^{2}+l0*p_{c}*whitevol^{2}\\ \;-\surd ((-2*l0*p_{c}{}^{2}*redvol^{2}-K_{D}*p_{c}*redvol*whitevol-l0\\ \;*p_{c}*redvol*whitevol-2*l0*p_{c}{}^{2}redvol*whitevol-p_{c}\\ \;*redvol*t0*whitevol-K_{D}*whitevol^{2}-l0*p_{c}\\ \;*whitevol^{2}{)}^{2}-4(p_{c}{}^{2}*redvol^{2}+p_{c}*redvol*whitevol)(l0^{2}\\ \;*p_{c}{}^{2}*redvol^{2}+K_{D}*l0*p_{c}*redvol*whitevol+2*l0^{2}*p_{c}{}^{2}\\ \;*redvol*whitevol+l0*p_{c}*redvol*t0*whitevol+K_{D}*l0\\ \;*p_{c}*whitevol^{2}+l0^{2}*p_{c}{}^{2}whitevol^{2}+l0*p_{c}*t0\\ \;*whitevol^{2}))) \end{array}$$

(5) $$\begin{array}{l} lwhite=\frac{1}{2\left(p_{c}{}^{2}*redvol+p_{c}*whitevol\right)}*(-K_{D}*p_{c}*redvol+l0*p_{c}\\ \;*redvol-p_{c}*redvol*t0-K_{D}*whitevol+l0*p_{c}\\ \;*whitevol+\surd (-4\left(-K_{D}*l0*redvol-K_{D}*l0*whitevol\right)({p_{c}}^{2}\\ \;*redvol+p_{c}*whitevol)\\ \;+(K_{D}*p_{c}*redvol-l0*p_{c}*redvol+p_{c}*redvol*t0+K_{D}\\ \;*whitevol-l0*p_{c}*whitevol)2)) \end{array}$$

(6) $$p_{t}=\frac{1}{2*K_{D}*redvol}*(\begin{array}{c} K_{D}*p_{c}*redvol-l0*p_{c}*redvol\\ +p_{c}*redvol*t0-K_{D}*whitevol-l0*p_{c}*whitevol\\ +\sqrt{\begin{array}{c} {\left(\begin{array}{c} -K_{D}*p_{c}*redvol+l0*p_{c}*redvol-p_{c}\\ {}*redvol*t0+K_{D}*whitevol+l0*p_{c}*whitevol \end{array}\right)}^{2}-\\ 4*K_{D}*redvol\left(\begin{array}{c} -l0*p_{c}{}^{2}*redvol-K_{D}*p_{c}*whitevol\\ -l0*p_{c}{}^{2}*whitevol-p_{c}*t0*whitevol \end{array}\right) \end{array}} \end{array})$$

**Figure 1:**
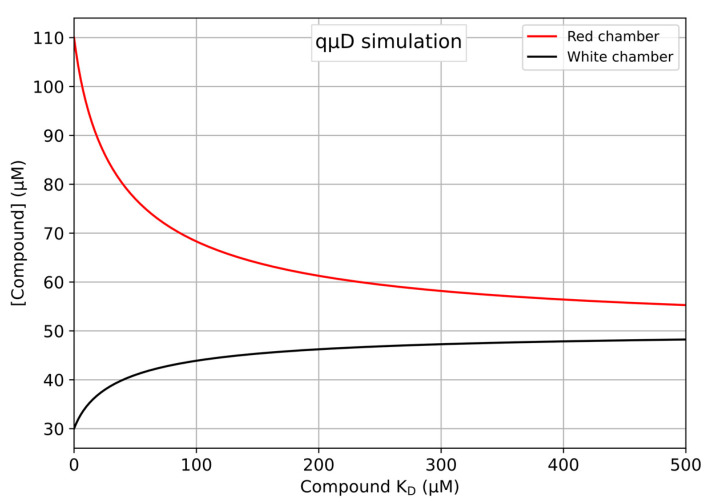
Protein–ligand complex K_D_ vs. compound concentration in the red and white chambers. Standard microdialysis system: 100 µL red chamber, 300 µL white chamber, [t0] = 80 µM and [l0] = 50 µM. Distinguishing between high K_D_s becomes difficult as K_D_s increase as concentrations converge and gradients reduce. Maximum sensitivity and lowest error in K_D_ determination will be present at low K_D_s (high affinities).

With the simulation of concentrations fully defined, the equations for K_D_ determination can be derived, which also contribute to experimental planning aspects. There are three equations for K_D_ determination, either by the measured concentration in the white chamber (*lwhite*), the red chamber (*lred*) or a combination of both (expressed as *p_t_*). Whilst simulation and production of concentration plots as shown in Figure 1 would allow determination of K_D_ from *y*-axis concentrations and *x*-axis K_D_ values, direct analytical solutions for K_D_ determination are quicker and more accurate. We may solve the quantitative microdialysis model previously defined for K_D_ in terms of *lred*, *lwhite* and *p_t_*, producing three equations for the determination of K_D_.

(7) $$\begin{array}{l} Solve\left[Simplify\left[Eliminate\left[model,tf,tl,lfred,lwhite,lred\right]\right],K_{D}\right]\\ Solve\left[Simplify\left[Eliminate\left[model,tf,tl,lfred,lwhite,pt\right]\right],K_{D}\right]\\ Solve\left[Simplify\left[Eliminate\left[model,tf,tl,lfred,lred,ptvalue\right]\right],K_{D}\right] \end{array}$$

Producing the following equations for K_D_:

(8) $$K_{D}{}^{from\;p_{t}}=\frac{\begin{array}{c} -l0*p_{c}{}^{2}redvol+l0*p_{c}*p_{t}*redvol\\ -p_{c}*p_{t}*redvol*t0\\ -l0*p_{c}{}^{2}whitevol+l0*p_{c}\\ {}*p_{t}*whitevol-p_{c}*t0*whitevol \end{array}}{\left(p_{c}-p_{t}\right)\left(p_{t}*redvol+whitevol\right)}$$

(9) $${K_{D}}^{from\;lred}=\frac{\left(\begin{array}{c} -l0^{2}*p_{c}{}^{2}*redvol^{2}+2*l0*lred*p_{c}{}^{2}*redvol^{2}-lred^{2}*p_{c}{}^{2}*redvol^{2}\\ +l0*lred*p_{c}*redvol*whitevol\\ -lred^{2}*p_{c}*redvol*whitevol-2*l0^{2}*p_{c}{}^{2}*redvol*whitevol\\ +2*l0*lred*p_{c}{}^{2}*redvol*whitevol-l0*p_{c}*redvol*t0*whitevol\\ +lred*p_{c}*redvol*t0*whitevol+l0*lred*p_{c}*whitevol^{2}\\ -l0^{2}*p_{c}{}^{2}*whitevol^{2}-l0*p_{c}*t0*whitevol^{2} \end{array}\right)}{\left(whitevol*\left(\begin{array}{c} l0*p_{c}*redvol-lred*p_{c}*redvol\\ -lred*whitevol+l0*p_{c}*whitevol \end{array}\right)\right)}$$

(10) $${K_{D}}^{from\;lwhite}=-\frac{\begin{array}{c} lwhite*(l0*p_{c}*redvol-lwhite*p_{c}{}^{2}*redvol\\ -p_{c}*redvol*t0+l0*p_{c}*whitevol-lwhite*p_{c}*whitevol) \end{array}}{l0*redvol-lwhite*p_{c}*redvol+l0*whitevol-lwhite*whitevol}$$

These direct analytical solutions for K_D_ allow analysis of experimental data in terms of the compound concentration in either chamber, or the ratio between them.

## 3. Procedure

### 3.1. Step 1: Obtaining Calibration Curves

1. Run a RP-HPLC analysis of the small molecules under investigation at 200 µM concentration, using the method above to determine the purity and retention time (Rt) of the compound. Additionally, identifying the absorption wavelength that gives the strongest absorption and most stable baseline. We found that this is usually the optical density (OD) at 210 nm or at 260 nm. Alternatively, for LC–MS, we ran an analysis of the compound at 5 µM using the method above with an injection volume of 2 µL. We determined the Rt and the mass of the protonated molecular ion of the compound [M+H]^+^ in the ion chromatogram, with M being the monoisotopic mass of the compound.
2. Create a series of dilutions of the small molecules to be tested in the assay buffer with 5% DMSO and 0.1% Pluronic f127 in HPLC insert vials, giving a total volume per concentration of 100 µL. The concentrations needed to depend on each individual compound. However, we found that a versatile initial choice of concentrations, applicable to most compounds, is 10, 25, 50, 75 and 100 μM. If LC–MS is to be used instead, a series of dilutions of 50, 100, 500, 1000 and 1500 nM are recommended, in an assay buffer with 5% DMSO in HPLC insert vials, at a total volume per concentration of 50 µL. Pluronic f127 is omitted in this instance as detergents ionise very well so can cause problems in mass spectrometry.
3. Run RP-HPLC or LC–MS analyses of the dilution series of compounds, in triplicate, using the method described above. Injection volume 25 µL (RP-HPLC) or 2 µL (LC–MS) for each concentration.
4. Analyse the data. For RP-HPLC, determine the area under the elution peak at the predetermined Rt in the particular chromatogram chosen in 3.1.1, and plot peak area versus each of the applied small molecule concentrations in triplicate. The graph should show a straight line if the analysis was performed with compound concentrations in the linear range of the detector. Based on our own analyses, we concluded that the linear range of peak area versus concentration of > 90% of small molecules tested covered a range between 25 and 200 µM. Fit the data to a linear y = kx + d equation using a suitable software (Excel, Origin, GraFit, etc.). These preanalysis runs will serve as reference information for the expected peak area for each test compound concentration. Alternatively for LC–MS, we obtained the ion chromatogram of the compound and determine the area under the [M+H]^+^ mass ion peak at its Rt, which was identified in 3.1.1. This ion peak area represents the total ion count; we plotted this against each of the small molecule concentrations in triplicate and followed the same procedure as for HPLC above to obtain a calibration curve. Based on our analyses, the linear range of our LC–MS covered a range between 100 and 1000 nM.

### 3.2. Step 2: Identification of Appropriate Target Protein Concentration

The simulation of concentrations present over a range of K_D_s is performed by using either the accompanying Python code or Microsoft Excel-based workbook; both are available at https://github.com/stevenshave/microdialysis. Alternatively, the equations derived in Section 2.3 (computational design–simulating system behaviour) may be translated into a preferred plotting or simulation environment. Working in a standard unit scale such as micro- or nano- simplifies the input and reduces the risk of numerical instability. Therefore, micromolar and microliter units were chosen as defaults for the input, calculations and output evaluation. Described below are the steps necessary to simulate a ‘standard’ HPLC-analysis-based microdialysis system using an 80 µM protein, a starting ligand concentration of 200 µM, which equilibrates to 50 µM in the absence of protein, and standard red and white volumes of 100 and 300 µL respectively. Target–ligand K_D_s from (fractionally above) 0 to 500 µM were simulated. These simulations served to ensure that unbound compound concentrations achieved at the K_D_s of interest were accurately readable with the equipment used.

Simulation using the Python code:

1. Open the Python program entitled “02_plot_KDvsConcentrations.py” in a text editor and assign simulation concentrations to the following variables:
  a. l0 = 50 → Compound (ligand) concentration achieved at equilibrium in both chambers in the absence of the protein. A starting concentration of 200 µM in the red chamber would equilibrate to 50 µM in both chambers in a system with standard volumes of 100 and 300 µL for the red and white chambers respectively.
  b. t0 = 80 → Protein (target) concentration in the red chamber.
  c. redvol = 100 → Volume of the red chamber.
  d. whitevol = 300 → Volume of the white chamber.
  e. KD_beginning = 0 → Lower boundary of the K_D_ range for the *X*-axis.
  f. KD_end = 500 → Upper boundary of the K_D_ range for the *X*-axis.
  g. pc = 1.0 → *p_c_* = 1 indicates that the compound equilibrates perfectly in the control qµD run.
2. Save and run the newly edited Python program to produce a plot as shown in Figure 1.
3. To perfom single point accurate measurements and read out the data, edit the Python program “01_simulate_qud_concentrations.py” and set l0, t0, red and whitevol as defined in the Python part of Section 3.2, additionally, set kdtl to be the precise value of K_D_ at which to read out the red and white volume concentrations.

Simulation using the Excel workbook:

1. Open the Excel workbook file entitled “quDSimulation_v1.xlsx” in Microsoft Excel, LibreOffice calc or a similar compatible spreadsheet program. Fields that should be edited by users to simulate a system are coloured red, and readout cells are green.
2. Open the sheet entitled ‘Simulation’ and edit the following fields as shown below to simulate the standard qµD system defined above. The plot produced automatically updates upon eachvariable manipulation.
  a. l0 = 50 → Compound (ligand) concentration achieved at equilibrium in both chambers in the absence of the protein. A starting concentration of 200 µM in the red chamber would equilibrate to 50 µM across chambers in a system with standard volumes of 100 and 300 µL for red and white chambers respectively.
  b. t0 = 80 → Protein (target) concentration in the red chamber.
  c. redvol = 100 → Volume of the red chamber.
  d. whitevol = 300 → Volume of the white chamber.
  e. KD_end = 500 → Upper boundary of the K_D_ range for the *X*-axis.
  f. pc = 1.0 → *p_c_* = 1 indicates that the compound equilibrates perfectly in the control qµD run.
3. The updated plot is displayed automatically on the sheet, producing the plot shown in Figure 1.
4. To perfom accurate single-point measurements and read out the data, select the ‘KD to conc+pValue’ sheet and fill in the same variables as listed in the Excel part of Section 3.2, adding a new value for ‘KD’ indicating the K_D_ at which to read out the red and white chamber ligand concentrations.

### 3.3. Step 3: Compound-only Dialisability Test

1. Define the compound concentration to use based on the peak area as a function of the concentration analysis performed in 3.1.4; it should lie in the middle of the linear range determined in 3.1.4. Keep in mind that after full equilibration only 25% of the starting concentration will be detectable (see qµD process outline above). For example, if the centre of the linear range found is 50 µM for the RP-HPLC analysis, then one needs to use a starting compound concentration of 200 µM. This is a direct consequence of the volumes present in the RED device.
2. Prepare at least 900 µL of the assay buffer. When RP-HPLC is used for analysis, this includes 5% DMSO and 0.1% pluronic f127. For LC–MS, Pluronic f127 is omitted.
3. To produce the standard qµD system modelled in Section 3.2, step 2, prepare a 350 µL solution of test compounds at 200 µM in the assay buffer with 5% DMSO and 0.1% pluronic f127. Apply sonication and vortexing to support compound solubilisation, and visually inspect the solution for precipitation. For LC–MS, prepare a 350 µL solution of test compounds at 40 µM in the assay buffer with 5% DMSO and use the same methods as above to make sure that the compound is fully in solution.
4. Clean the base plate thoroughly with 20% ethanol and allow it to dry.
5. Place 3 RED devices into the base plate for each compound to be tested.
6. Add 100 µL of compound solution into the red chamber of each RED device and 300 µL of assay buffer with 5% DMSO and 0.1% pluronic f127 in the white chamber, respectively. For LC–MS: add 100 µL of compound solution into the red chamber of each RED device and 300 µL of assay buffer with 5% DMSO.
7. Seal the base plate with a self-adhesive plate seal to inhibit evaporation.
8. Place the base plate onto an orbital shaker at 250 rpm for 6 h for equilibration. Up to 6 h is the suggested time by the manufacturers but this needs to be investigated for the compound under study; some compounds may require an overnight equilibration.
9. After > 6 h equilibration time, harvest 50 µL from each chamber of each RED tube and run RP-HPLC using the identical gradient and 25 µL injection volume. If fully equilibrated, the compound concentration in either chamber should be 50 µM. For LC–MS, harvest 10 µL from each chamber of the RED tube, and dilute 20 × with 0.1% formic acid solution. Run the diluted solution using the LC–MS method above, injection volume 2 µL. If fully equilibrated, the compound concentration in either chamber should be 10 µM and after 20 × dilution it should be 500 nM.
10. Check that the Rt in the chromatographic elution profile of each test sample has not changed from the condition-setting experiments. Determine the peak area for the compounds in the solutions taken from the red and the white chamber for each HPLC run. Determine *p_c_* from the ratio of these two peak areas. A well-equilibrated compound should result in a *p_c_* of 0.90–1.10. Any compound showing larger than 10% deviation from 1.00 should be considered for exclusion from further analysis. For LC–MS, check that the Rt in the ion chromatogram of each sample has not changed from the condition setting experiments. Determine the mass ion peak area for the compounds in the solutions taken from the red and white chamber for each LC–MS run. Perform a data analysis as above using this total ion count.
11. Additionally, if a compound shows full equilibration with 0.90 < *p_c_* < 1.10, check for compound losses, which could occur for a variety of reasons described in the introduction section. If the area under the chromatographic peaks (or mass ion peak in LC–MS) is within 10% of the one found during the initial calibration experiment using a 50 µM (500 nM for LC–MS) concentration, it can be assumed that compound losses are negligible.

### 3.4. Step 4: Microdialysis Target Protein–Small Molecule Binding assay

1. Place 6 RED devices (3 RED devices containing compounds only, and 3 containing compounds + target protein) into the cleaned base plate.
2. Prepare 2 mL of assay buffer with 5% DMSO and 0.1% pluronic f127 when HPLC is used for analysis. When LC–MS is used for analysis, prepare 2 mL of the assay buffer containing 5% DMSO.
3. Prepare 350 µL of 200 µM small molecule solution in the assay buffer with 5% DMSO and 0.1% pluronic f127, and 350 µL of 200 µM small molecule plus 80 µM protein in the same buffer. For LC–MS, prepare 350 µL of 40 µM small molecule solution in the assay buffer with 5% DMSO and 350 µL of 40 µM small molecule plus 40 µM protein in the same buffer.
4. Add 3 × 100 µL of 200 µM (or 40 µM) compound solution to 3 red chambers of the RED tubes followed by adding 3 × 300 µL dialysis buffer in the corresponding white chambers.
5. Add 3 × 100 µL of 200 µM + 80 µM protein (or 40 µM compound + 40 µM protein) solution into 3 red chambers of the RED tubes and add 3 × 300 µL of dialysis buffer in the corresponding white chambers.
6. Seal plate with the self-adhesive plate seal and place on shaker at 250 rpm for 6 h.
7. After 6 h or overnight incubation, remove the plate from the shaker and harvest 50 µL each from the red and white chamber of the compound-only samples, as well as 50 µL from the white chambers of the compound-protein binding samples; and add them to insert vials for the HPLC analysis. For the LC–MS analysis, take out 10 µL from each red and white chamber of the compound alone samples as well as from the compound and protein samples.
8. Run these samples on RP-HPLC under the same conditions as above, with an injection volume of 25 µL, or for LC–MS, dilute the 10 µL aliquot 20 × with 0.1% FA solution and run the LC–MS analysis, injection volume 2 µL.
9. From the resulting chromatograms, obtain peak areas at known Rts for the samples for RP-HPLC. For LC–MS, obtain mass ion peak areas of the compounds at known Rts.

For compound-only experiments:

10. Determine *p_c_* = compound peak area from red chamber/white chamber or determine *p_c_* = mass ion peak area from red chamber/white chamber.
11. This should resemble the data achieved from the compound equilibration test experiments and should therefore be close to 1.0.
12. For determination of the total amount of compounds in the qµD. Convert the compound peak area or mass ion peak area obtained in Section 3.3. 11 into compound concentration by using the calibration curve in Section 3.1. 4. The amount of compound in the chambers can be obtained by multiplication of the converted compound concentration in the red chamber by 100 µL plus the converted compound concentration of the white chamber multiplied by 300 µL. This should yield close to the amount of introduced compound at the start of the dialysis (200 µM × 100 µL = 20 × 10^−9^ mol for HPLC and 40 µM × 100 µL = 4 × 10^−9^ mol for LC–MS) indicating no losses due to adhesion, precipitation or aggregation effects. Note that for LC–MS the solutions were diluted 20 × before analysis. Therefore, one needs to multiply by a factor of 20 to obtain the real concentrations in the RED tube.

For compound-target protein binding experiments:

13. From the HPLC chromatograms of the white chambers, determine the peak area and convert to compound concentration by using the calibration curve above.
14. Determine the amount of compound in the white chamber by multiplication of the determined compound concentration with the volume of the white chamber, 300 µL.
15. From Section 3.3. 13 the total amount of compound is known. Therefore, the amount of compound in the red chamber is the difference between the total amount of compound and the amount of compound in the white chamber.
16. Calculated the compound concentration in the red chamber by dividing the amount of compound in the red chamber by 100 µL.
17. Determine *p_t_* by dividing the compound concentration of the red chamber by that of the white chamber. For LC–MS, determine *p_t_* by dividing the compound mass ion peak area of the red chamber by that of the white chamber obtained in Section 3.3. 9.

### 3.5. Step 5: Use the Software to Determine the Dissociation Constant (K_D_) of the Compound to the Target Protein by Using the Red/White Chamber Concentrations or p_t_ Values

Determination of the dissociation constants (K_D_s) can be performed by either using the accompanying Python code or Microsoft Excel-based workbook, both available at https://github.com/stevenshave/microdialysis. Below we performed the steps necessary to determine a K_D_ for a ‘standard’ HPLC-analysis-based microdialysis system using 80 µM protein, a starting ligand concentration of 200 µM, equilibrating to 50 µM in the absence of protein and standard red and white volumes of 100 and 300 µL respectively. There were three equations for K_D_ determination, either by the measured concentration in the white chamber (*lwhite*), the red chamber (*lred*), or a combination of both, as expressed as *p_t_*.

K_D_ determination using the Python code on single point measurement data:

1. To determine a K_D_ based on only a single point measurement of the white chamber concentration, open the Python file entitled ‘03_deriveKD_from_lwhite.py’ in a text or code editor and assign the following variables, taking care to input the correct value for lwhite as the measured concentration of ligand in the white chamber and run the program to read out the K_D_:
  - l0 = 50, Compound (ligand) concentration achieved at equilibrium in both chambers in the absence of the protein. A starting concentration of 100 µM in the red chamber would equilibrate to 25 µM across chambers in a system with standard volumes of 100 and 300 µL for red and white chambers respectively.
  - t0 = 80, Protein (target) concentration present in the red chamber.
  - redvol = 100, Volume of the red chamber.
  - whitevol = 300, Volume of the white chamber.
  - lwhite, The ligand concentration as measured in the white chamber.
  - pc, *p_c_* = 1 indicates that the compound equilibrates perfectly in the control qµD run.
2. To determine a K_D_ based on only a single point measurement of the red chamber concentration, open the Python file entitled ‘03_deriveKD_from_lred.py’ in a text or code editor and assign the following variables, taking care to input the correct value for lred as the measured concentration of ligand in the red chamber and run the program to read out the K_D_:
  - l0 = 50, Compound (ligand) concentration achieved at equilibrium in both chambers in the absence of the protein. A starting concentration of 100 µM in the red chamber would equilibrate to 25 µM across chambers in a system with standard volumes of 100 and 300 µL for red and white chambers respectively.
  - t0 = 80, Protein (target) concentration present in the red chamber.
  - redvol = 100, Volume of the red chamber.
  - whitevol = 300, Volume of the white chamber.
  - lred, The ligand concentration as measured in the red chamber.
  - pc, *p_c_* = 1 indicates that the compound equilibrates perfectly in the control qµD run.
3. To determine a K_D_ based on a *p_t_* derived from single point measurements open the Python file entitled ‘03_deriveKD_from_pt.py’ in a text or code editor and assign the following variables, taking care to input the correct value for pt as the ratio of compound concentration the the red versus white chambers and run the program to read out the K_D_:
  - l0 = 50, Compound (ligand) concentration achieved after equilibrium in both chambers in the absence of the protein. A starting concentration of 100 µM in the red chamber would equilibrate to 25 µM across chambers in a system with standard volumes of 100 and 300 µL for red and white chambers respectively.
  - t0 = 80, Protein (target) concentration present in the red chamber.
  - redvol = 100, Volume of the red chamber.
  - whitevol = 300, Volume of the white chamber.
  - pt, The *p_t_* value, which is the concentration of ligand in the red chamber divided by the concentration of ligand in the white chamber.
  - pc, *p_c_* = 1 indicates that the compound equilibrates perfectly in the control qµD run.

K_D_ determination using the Python code on multiple replicates of measurement data:

1. To determine a K_D_ based on multiple replicate measurements of compound concentration in the white chamber, open ‘04_deriveKD_from_multiple_lwhite.py’ in a text or code editor. Comments in the file provide instructions in line with the following:
  - Assign the experimental variables as descibed in Section 3.5. 1 above, setting l0, t0, redvol and whitevol.
  - If 3 measurements of compound concentration lwhite have been made, calculate the mean and standard deviation of these variables using numpy (val1, val2 and val3 bellow are measurement replicates):
    - lwhite_mean = np.mean([val1, val2, val3]);
    - lwhite_std = np.std([val1, val2, val3]).
  - Assign lwhite to be a newly constructed Python uncertainties package ufloat object.
    - lwhite = ufloat(lwhite_mean, lwhite_std).
  - If multiple *p_c_* values have been derived, the pc variable may be assigned to a ufloat object constructed as:
    - pc_mean = np.mean([val1, val2, val3]);
    - pc_std = np.std([val1, val2, val3]);
    - pc = ufloat(pc_mean, pc_std).
  - Alternatively, a single *p_c_* value may be assigned as in 3.5.
2. To determine a K_D_ based on multiple replicate measurements of compound concentration in the red chamber, open ‘04_deriveKD_from_multiple_lred.py’ in a text or code editor. Comments in the file provide instructions in line with the following:
  - Assign the experimental variables as descibed in Section 3.5. 1 above, setting l0, t0, redvol and whitevol.
  - If 3 measurements of compound concentration lred have been made, calculate the mean and standard deviation of these variables using numpy (val1, val2 and val3 bellow are measurement replicates):
    - lred_mean = np.mean([val1, val2, val3]);
    - lred_std = np.std([val1, val2, val3]).
  - Assign lwhite to be a newly constructed Python uncertainties package ufloat object.
    - lred = ufloat(lred_mean, lred_std).
  - If multiple *p_c_* values have been derived, the pc variable may be assigned to a ufloat object constructed as:
    - pc_mean = np.mean([val1, val2, val3]);
    - pc_std = np.std([val1, val2, val3]);
    - pc = ufloat(pc_mean, pc_std).
  - Alternatively, a single *p_c_* value may be assigned as in Section 3.5. 1.
3. To determine a K_D_ based on multiple replicate measurements of *p_t_*, open ‘04_deriveKD_from_multiple_pt.py’ in a text or code editor. Comments in the file provide instructions in line with the following:
  - Assign the experimental variables as descibed in Section 3.5. 1 above, setting l0, t0, redvol, and whitevol.
  - If 3 measurements of *p_t_* have been made, calculate the mean and standard deviation of these variables using numpy (val1, val2 and val3 bellow are measurement replicates):
    - pt_mean = np.mean([val1, val2, val3]);
    - pt_std = np.std([val1, val2, val3]).
  - Assign lwhite to be a newly constructed Python uncertainties package ufloat object.
    - pt = ufloat(pt_mean, pt_std).
  - If multiple *p_c_* values have been derived, the pc variable may be assigned to a ufloat object constructed as:
    - pc_mean = np.mean([val1, val2, val3]);
    - pc_std = np.std([val1, val2, val3]);
    - pc = ufloat(pc_mean, pc_std).
  - Alternatively, a single *p_c_* value may be assigned as in Section 3.5. 1.

K_D_ determination using the Excel workbook code:

1. To determine a K_D_ based on only the white chamber concentration, open the Excel file entitled ‘quDSimulation_v1.xlsx’ in Microsoft Excel, LibreOffice calc, or similar compatible spreadsheet program. Go to the tab named ‘lwhite to KD’ and assign the following variables, taking care to input the correct value for lwhite as the measured concentration of ligand in the white chamber. The readout of K_D_ will update with every change of input variable:
  - l0 = 50 → Compound (ligand) concentration achieved at equilibrium in both chambers in the absence of the protein. A starting concentration of 100 µM in the red chamber would equilibrate to 25 µM across chambers in a system with standard volumes of 100 and 300 µL for red and white chambers respectively.
  - t0 = 80 → Protein (target) concentration present in the red chamber.
  - redvol = 100 → Volume of the red chamber.
  - whitevol = 300 → Volume of the white chamber.
  - lwhite → The ligand concentration as measured in the white chamber.
  - pc → *p_c_* = 1 indicates that the compound equilibrates perfectly in the control qµD run.
2. To determine a K_D_ based on only the red chamber concentration, open the Excel file entitled ‘quDSimulation_v1.xlsx’ in Microsoft Excel, LibreOffice calc, or similar compatible spreadsheet program. Go to the tab named ‘lred to KD’ and assign the following variables, taking care to input the correct value for LRED as the measured concentration of ligand in the red chamber. The readout of KD will update with every change of input variable:
  - l0 = 50 → Compound (ligand) concentration achieved at equilibrium in both chambers in the absence of the protein. A starting concentration of 100 µM in the red chamber would equilibrate to 25 µM across chambers in a system with standard volumes of 100 and 300 µL for red and white chambers respectively.
  - t0 = 80 → Protein (target) concentration present in the red chamber.
  - redvol = 100 → Volume of the red chamber.
  - whitevol = 300 → Volume of the white chamber.
  - lred → The ligand concentration as measured in the red chamber.
  - pc → *p_c_* = 1 indicates that the compound equilibrates perfectly in the control qµD run.
3. To determine a K_D_ based on *p_t_*, open the Excel file entitled ‘quDSimulation_v1.xlsx’ in Microsoft Excel, LibreOffice calc or a similar compatible spreadsheet program. Go to the tab named ‘pt to KD’ and assign the following variables, taking care to input the correct value for PT_VALUE as the measured and calculated *p_t_* value. The readout of KD will update with every change of input variable:
  - l0 = 50 → Compound (ligand) concentration achieved at equilibrium in both chambers in the absence of the protein. A starting concentration of 100 µM in the red chamber would equilibrate to 25 µM across chambers in a system with standard volumes of 100 and 300 µL for red and white chambers respectively.
  - t0 = 80 → Protein (target) concentration present in the red chamber.
  - redvol = 100 → Volume of the red chamber.
  - whitevol = 300 → Volume of the white chamber.
  - pt → The *p_t_* value, which is concentration of the ligand in the red chamber divided by the concentration of the ligand in the white chamber.
  - pc → *p_c_* = 1 indicates that the compound equilibrates perfectly in the control qµD run.

## 4. Expected Results

### 4.1. Simulation of the qµD Experiment as a Function of the Concentrations and K_D_s

With equations established to simulate the concentration of compound present in each chamber, the expected red and white chamber concentrations can be evaluated in relation to the sensitivity of the chosen instruments. Since K_D_ determination is achieved by the accurate comparison of compound concentrations in the two chambers, or by inferring either one from the other in the case of only using one concentration, it is important that the experimental setup is such that a sensitive quantitative and accurate concentration determination is possible over the range of conditions of most interest. This may direct the experimental design to search for high or low affinity hits in screening campaigns and directly influences assay sensitivity, threshold values and Z’ values as an assessment of assay quality.

Figure 1 demonstrates how compound concentrations in each of the microdialysis chambers converged as the K_D_ increased. The simulation was performed as step 2 of the protocol and is necessary to understand the range of K_D_s resolvable as a function of concentrations and the accuracy of the instruments used to measure them. The simulation shows a typical setup for readout via HPLC, in which 80 µM of target protein was placed into the red chamber, along with an amount of ligand, which in the absence of the protein would equilibrate to 50 µM. Large separation of concentrations at low K_D_s (high affinity) allowed for a confident and accurate assignment of K_D_ from concentrations. At the upper end of the simulated K_D_ range between 400 and 500 µM, the concentration differed minimally, making a direct assignment of K_D_ within this range less accurate. This difficulty in confidently assigning high K_D_s is further demonstrated in Scheme 1, Step 5, whereby *p_t_* is calculated and shown to be converging to what would be *p_c_*.

**Figure 2:**
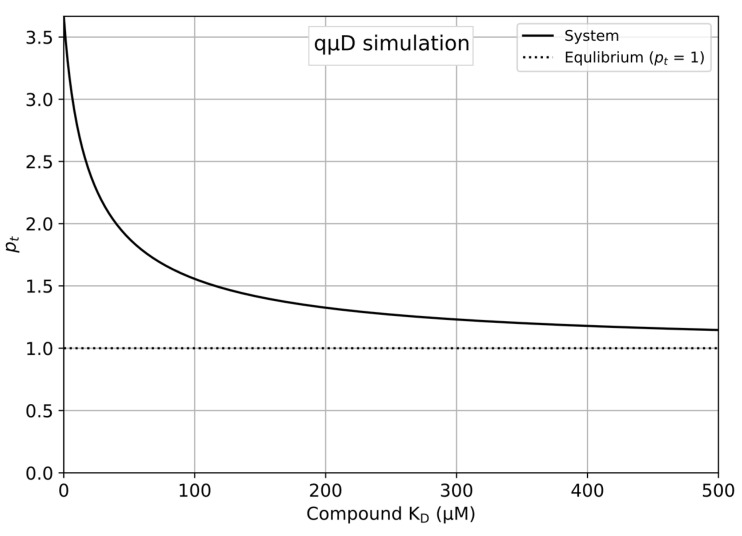
The protein–ligand complex K_D_ vs. the *p_t._* standard microdialysis system, 100 µL red chamber, 300 µL white chamber, [t0] = 80 µM and [l0] = 50 µM. At higher concentrations, the ratio of the compound within each chamber reaches equilibrium as the *p_t_* value becomes asymptotic to *p_t =_* 1. Very precise concentration measurements would be needed to accurately determine high K_D_s or identify them as hit compounds.

Figure 2 further demonstrated the difficulty in using *p_t_* to assign high K_D_s, with the K_D_s of 300 and 500 µM producing *ptvalues* of 1.247 and 1.153 respectively, *lred* values of 29.366 and 27.760 respectively and *lwhite* values of 23.545 and 24.081 µM, respectively.

### 4.2. Exemplaric Considerations on the Accuracy of K_D_ Determinations

Differentiating the three equations for K_D_ with respect to *lred*, *lwhite* and *p_t_* allows investigation of the rate of change of K_D_ as a function of changing concentrations, further illustrating the importance of accurate concentration determination. Using the standard microdialysis system simulated in Figure 1 and Figure 2, where *p0* was 80 µM, *l0* was 50 µM and the standard volumes of 100 and 300 µL for the red and white chambers, differentiation with respect to *lred*, *lwhite* and *p_t_* shows gradients along with concentrations and *p_t_* values present at intervals between 1 and 500 µM. From these values given in Table 1, we clearly see the instantaneous gradient present at red and white concentrations was smaller at K_D_ levels of 1 vs. 500 µM K_D_ values. A unit change in red or white concentration invoking almost a 200-fold instantaneous change in K_D_ at 500 vs. 1 µM K_D_s. As the change in the K_D_ value as calculated by *p_t_* was so large, it was suggested that only one singular concentration be used for K_D_ determination. Ideally this would be the red chamber, which had the smallest gradient with respect to the compound concentration present across all K_D_ values. However, with protein present, steps for compound concentration determination were further extended with the removal of the protein from samples. A general rule is therefore suggested whereby the white chamber compound concentration (*lwhite*) was used for K_D_ determination. The relationship between *lred* and *lwhite* for perfectly equilibrating compounds (*p_c_* = 1.00) shows identical fold-changes in gradient across K_D_s compared to a baseline gradient at a K_D_ of 1 µM. We concluded that the qµD method was best suited for determination of K_D_s less than 200 µM.

**Figure 3:**
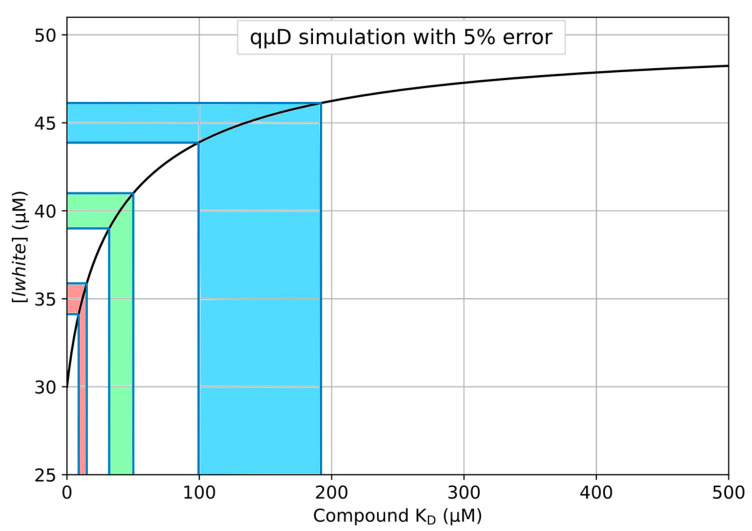
K_D_ determination using a 5% concentration error. Standard microdialysis system, 100 µL red chamber, 300 µL white chamber, [t0] = 80 µM and [l0] = 50 µM. *lwhite* compound concentrations of 35 (red), 40 (green) and 45 µM (blue) give rise to K_D_ ranges of 8.9–14.9 µM, 31.9–50.1 µM and 99.4–191.9 µM respectively.

Figure 3 illustrates the possible K_D_ range calculated using a detection method and handling technique, which introduced a ±5% (measured at the 2 σ confidence level) error. The valid 2σ K_D_ ranges for the standard HPLC example system used above, with *lwhite* concentrations of 35, 40 and 45 µM were: 8.9–14.9 µM, 31.9–50.1 µM and 99.4–191.9 µM, respectively.

An accurate determination of K_D_s will always depend on the accuracy of the concentration determination, which is linked to instrument sensitivity and handling. The impact of these errors may be minimised through proper planning and simulation, knowing the K_D_ ranges of most interest and setting rejection criteria for hit K_D_s. The mathematical treatment of the microdialysis system as defined in this protocol also increases the accuracy of the technique compared to the previously published simplified equations, which rely on the assumption that the amount of free protein is in great excess of the bound protein. Our protocol does not include such assumptions and represents a correct treatment of the system.

System simulation and reproduction of all plots may be achieved using the supplied Python code or the included Microsoft Excel workbook at https://github.com/stevenshave/microdialysis.

### 4.3. Experimental Results

In the following we summarized the outcome of two qµD experiments, which were performed with primary hits from a plate-based size-exclusion chromatography screen [10] against two validated human cancer targets and one antibacterial target. This method allowed the identification of low affinity binders in the low mM K_D_ range. However, quantification was only relative and exact affinity determination of the target was cumbersome. Additionally, compounds that aggregate in solution at the assay concentrations can result in false positives, since they are spun through a size exclusion membrane as fast as if they were bound to the target protein. Therefore, qµD was applied to check the aggregation and surface binding properties of the primary hit compounds and if suitable, their target binding K_D_s. For compounds 1–3 the RP-HPLC analysis was applied, for compound 4 the analysis was performed using an LC–MS read out (Table 2).

After establishing the calibration curve via RP-HPLC and using the simulation to obtain a suitable protein concentration (t0 = 80 µM), the equilibration of compounds in the absence of the protein was performed to obtain a *p_c_* value from a 100 µM starting compound concentration (l0 = 25 µM). After a dialysis time of 6 h and an overnight incubation (16 h), the resulting *p_c_* value for compound 1 based on a decorated phenylpiperazine scaffold was significantly higher than the set threshold of 1.10. This compound, therefore, never reached equilibrium. Hence, it could not be used for K_D_ determination and was excluded from the hit to lead processes.

In contrast, compound 2 based on a pyridin-2-one scaffold equilibrates well with a *p_c_* of 1.02, reached within 6 h from a starting compound concentration of 100 µM and protein concentration of 80 µM in the red chamber (l0 = 25 µM). Unfortunately, when the experiment was performed with its target protein and BSA control, *p_t_* was less than *p_s_* suggesting that the compound binds to BSA with higher affinity than to its target protein with K_D_s of 278 ± 57 µM and 603 ± 154 µM respectively. Compound 3 based on a pyridazine scaffold also equilibrated well with *p_c_* = 1.02. In the presence of 80 µM of its target protein and a starting compound concentration of 100 µM (l0 = 25 µM), qµD yielded a *p_t_* = 1.21. The BSA binding test resulted in *p_s_* = 1.11, suggesting that although this compound demonstrated some low affinity binding with a calculated K_D_ of 882 ± 57 µM to BSA, it possessed a higher affinity to its target protein with a K_D_ of 405 ± 35 µM. The binding of compound 4 also containing a decorated phenylpiperazine scaffold had been validated previously using NMR. Therefore, further specificity controls against proteins such as BSA were not carried out at this stage. As seen in Table 2, the *p_c_* value was 0.99, when incubated with 40 µM protein and at a starting compound concentration of 40 µM (l0 = 10 µM), indicating that this compound was fully equilibrated after 16 h incubation. The *p_t_* value in the presence of its target protein was determined to be 1.21, which corresponded to a dissociation constant (K_D_) of 171 ± 34 µM; in strong agreement with the K_D_ of 154 ± 30 µM determined via 2D HSQC NMR titrations, with compound concentrations ranging between 0 and 2 mM.

## Figures and Tables

**Table 1:** Values for *lred*, *lwhite* and *p_t_*, and first derivatives thereof over a range of K_D_s for the standard system where *p0* is 80 µM, *l0* is 50 µM and the red and white chambers have standard volumes of 100 and 300 µL, respectively. A unit change in *lred* concentration at 55.3 µM (K_D_ of 500 µM) results in a 193-fold change in K_D_ readout compared to the 108 µM concentration (K_D_ 1 µM level). Similar changes are seen in the white chamber.

	*lred*		*lwhite*		*p_t_*	
K_D_	Concentration (µM)	∂K_D_/∂*lred* (Fold Change from 1 µM)	Concentration (µM)	∂lK_D_/∂*lwhite* (Fold Change from 1 µM)	Value	∂K_D_/∂*p_t_* (Fold Change from 1 µM)
1	108.1032	−0.5553 (1)	30.6323	1.6659 (1)	3.5291	−7.8158 (1)
100	68.304	−8.6209 (16)	43.8987	25.8628 (16)	1.5559	−249.2 (32)
200	61.2679	−23.2953 (42)	46.244	69.8859 (42)	1.3249	−747.259 (96)
300	58.1682	−44.6309 (80)	47.2773	133.8926 (80)	1.2304	−1496.34 (192)
400	56.4121	−72.6318 (131)	47.8626	217.8954 (131)	1.1786	−2495.81 (319)
500	55.2795	−107.299 (193)	48.2402	321.8969 (193)	1.1459	−3745.46 (479)

**Table 2:** Summary of results obtained with the qµD protocol on compounds, which showed low dialisability (1), nonspecific binding (2), strong specific and weak nonspecific binding (3) and a specific binder, which affinity to the target was compared to the affinity determined by NMR.

Compound #	[l0] (µM)	[t0] (µM)	*p_c_*	*p_t_*	*p_s_* (BSA)	Comment
1	25	80	1.41 ± 0.02	NA	NA	Non-equilibrating compound.
2	25	80	1.02 ± 0.01	1.15 ± 0.03	1.29 ± 0.05	Equilibrating compound, but it binds BSA stronger than to the target protein; 603 ± 154 µM vs. 278 ± 57 µM–assuming 1:1 binding.
3	25	80	1.02 ± 0.01	1.21 ± 0.01	1.11 ± 0.01	Equilibrating compound, K_D_ = 405 ± 35 μM to target protein, and 882 ± 57 µM to BSA–assuming 1:1 binding.
4	10	40	0.99 ± 0.03	1.21 ± 0.04	NA	Well-equilibrating compound, K_D_ = 171 ± 34 μM.
